# Origin, Impact, and Solutions for Lifestyle-Related Chronic Diseases in Samoa and American Samoa

**DOI:** 10.7759/cureus.17749

**Published:** 2021-09-05

**Authors:** Sable Neuendorf, Jadon Neuendorf, Mohsin Yakub

**Affiliations:** 1 Medical Education, California University of Science and Medicine, Colton, USA

**Keywords:** chronic disease, lifestyle medicine, nutrition, obesity, samoa and american samoa

## Abstract

Samoa and American Samoa are two island groups in the South Pacific inundated with the physiological consequences of swift westernization of diet and lifestyle. These polities face the singular theme of lifestyle-related problems seen in other countries. This paper aims to discuss the current demographics in Samoa and American Samoa and examine the origin and impact of lifestyle-related chronic diseases within a subset of its populace. This review will highlight the prominent nutrition transition that these polities have undergone in their development and examine the pathogenesis and pathophysiology of lifestyle-related diseases, primarily type 2 diabetes and obesity, in the context of a prominent cultural shift. Samoa and American Samoa face a litany of public health concerns as a result of the rising prevalence of lifestyle-related chronic diseases and the persistent threat of obesity. Lifestyle medicine is proposed as the optimal treatment solution for the currently devastating disease states and is adapted to the vibrant agricultural resources and healing roots of the Samoan culture.

## Introduction and background

The prevalence and rising incidence of lifestyle-driven chronic diseases plague the globe, topping the charts as the leading cause of death and a primary source of the global burden of healthcare costs [1]. The incidence and prevalence of chronic diseases are advancing across every region and pervading all socioeconomic classes. It is estimated that in 2020, 70% of all deaths worldwide are due to chronic diseases [1]. Conditions such as cardiovascular disease, type 2 diabetes, and obesity were not always this prevalent. To understand how and why Samoa and American Samoa have reached this situation, this paper aims to examine the rising incidence in these middle- and low-income regions undergoing nutrition transition as they develop. American Samoa and Samoa present a concentrated microcosm of accelerated westernization and development, which allows the visualization of the origin and impact of lifestyle-related diseases on a population. The purpose of this review is to describe the origin of, the impact of, and possible solutions to lifestyle-driven chronic diseases in American Samoa and Samoa.

**Figure 1:**
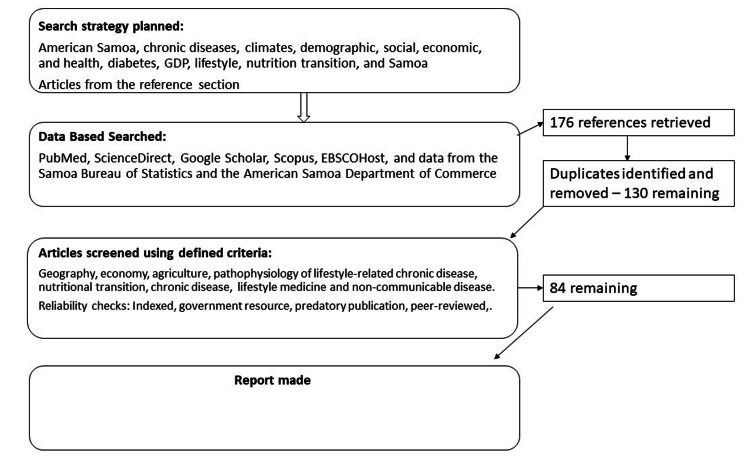
Flow chart of the review methodology.

Data used for this review included searches conducted in academic scientific databases, including PubMed, ScienceDirect, Google Scholar, Scopus, EBSCOHost, and data from the Samoa Bureau of Statistics and the American Samoa Department of Commerce. The following search terms were used: “American Samoa,” “chronic diseases,” “climates,” “demographics; social, economic, and health,” “diabetes,” “GDP,” “lifestyle,” “nutrition transition,” and “Samoa.” Additional articles were selected from the reference sections of articles that were reviewed. To procure sources regarding the science and pathophysiology of lifestyle-related chronic diseases, the reference section of the book “How Not to Diet” by Michael Greger M.D. FACLM was utilized. Lastly, to establish evidence-based recommendations, the American College of Lifestyle Medicine and the Samoa Ministry of Health were sourced. Figure 1 illustrates the flow chart of the review methodology.

## Review

Geography, economy, and agriculture

American Samoa and Samoa constitute a group of islands located south of the equator in the Pacific Ocean. American Samoa, lying just east of the international date line, includes seven islands with a total area of 77 square miles and a recently reported population of 55,519 (mostly residing on the island of Tutuila) [2]. Samoa lies just west of the international date line and includes 10 islands, four of which are inhabited with a total land area of 1,100 square miles and a recently reported population of 1,95,979. The islands of Upolu and Savai’i constitute the majority of land area and population [3].

In American Samoa, the two primary employment sources are the government sector and the tuna canning industry, yielding an estimated annual per-capita income of $14,000 [4,5]. The principal source of employment in Samoa is the agricultural industry, with an annual per-capita income of approximately one-third that of American Samoa ($4,067, adjusted to U.S. dollars) [6,7]. When comparing American Samoa to Samoa, higher disease rates have been associated with the higher annual per-capita income of individuals in American Samoa [8]. Over half of the population in both polities are unemployed, with many in Samoa choosing to be involved only in subsistence agriculture and living from their plantations [9,10]. In the 2017 Samoa Labour Force Survey, 94.6% (105,000) of the individuals surveyed participated in subsistence production [11]. In American Samoa, the percentage of the population engaged in subsistence agriculture significantly lowered as the land in farms decreased [12].

The archipelago experiences tropical weather year-round, with the hot, rainy season extending from December to April and the cool, dry season lasting from May to November [13]. Samoa and American Samoa are both rich in vibrant, fertile agriculture. In Samoa, forest land is more accessible and abundant than in the American Samoa island of Tutuila, where agricultural land is located on the incline of mountain slopes [14,15]. Crops grown in both American Samoa and Samoa include taro, ta’amu (giant taro), coconut, banana, yam, cocoa, and breadfruit [16,17].

With access to rich agriculture, the traditional Samoan diet was largely plant-based. Coconut products, starchy vegetables, leafy greens, fresh fruit, and saltwater fish were staples of the traditional Samoan diet, with chicken and pork present in lesser proportion and reserved for celebrations [18]. Other vegetables commonly consumed in Samoa included pumpkin, carrots, cabbage, and hibiscus greens [9]. Fruits such as banana and papaya, traditionally prepared in soups and coconut cream, were also dietary staples [19]. From the personal observation of one of the authors who spent two years living in American Samoa and Samoa, the foods from the traditional Samoan diet outlined by DiBello et al. were primarily seen in the rural, economically inactive population [19]. The daily diet in urban areas of both polities, where the population was more economically active, has tended to eliminate traditional, nutrient-dense food options and to substitute with imported, processed goods such as pastries, fried meats, and white rice [19,20]. Additionally, processed snack items such as chips, crackers, cakes, and sodas have become increasingly common [19].

Nutrition transition and physical activity

As low- and middle-income countries develop economically, a characteristic nutrition transition accompanies this growth. This transition is marked by the sharp increase in “consumption of vegetable oils, animal fats, and added sugars ... while the consumption of starchy staples declines” [21]. Imported high-calorie foods that line the market shelves are divergent from the traditional starchy staples of yam and taro, some of Samoa’s roots. The Harvard T.H. Chan School of Public Health outlined five patterns of a nutrition transition, with the fourth pattern being the abrupt shift to overeating and obesity-related diseases. This pattern was initiated by easy access to high-calorie foods and a shift to a more sedentary lifestyle, as was observed in American Samoa and Samoa [22].

In the last several decades, these polities have transitioned towards the replacement of subsistence agriculture with paid employment and have experienced an associated influx of imported foods [23]. Processed imports such as meats, vegetable oils, rice, cereals, pastries, and candy ushered in calorie-rich, nutrient-poor food sources such that the energy availability (or the availability of calories) per capita per day increased by 47% from 1961 to 2007 [23]. With easy access to craveable, energy-dense food sources, the population became reluctant to turn to their plantations where similar calorie counts would take months of planting, tending, and harvesting. This led to a concurrent decrease in manual labor and physical activity and a notable shift to a sedentary lifestyle. According to the noncommunicable disease (NCD) STEPwise approach to Surveillance (STEPS) report for population-based statistics on American Samoa’s physical activity, 61.7% of the study population was physically inactive [24]. In another study, Heard et al. reported that physical inactivity contributed to increasing NCD rates. Collectively, 21% of the Samoan population did very little or no physical activity, with rates of physical inactivity being higher in urban areas (28%) [25]. The physiological impacts of these lifestyle changes, compounded by the personal and social stressors that accompanied these rapid cultural changes, generated alarming chronic disease rates within these islands [26].

Chronic disease rates

In American Samoa and Samoa, the leading causes of mortality and morbidity are heart disease, diabetes, cancer, and stroke [27,28]. In Samoa, the leading cause of death is cardiovascular disease (34%), followed by other NCDs (18%), cancer (15%), and diabetes (9%) [27]. An estimated 81% of deaths in Samoa can be attributed to NCDs [27]. In American Samoa, 71.8% of the population is considered to be at high risk for NCDs, including risk factors such as smoking, obesity, hypertension, and low consumption of fruits and vegetables [28]. Due to its political ties and higher GDP per capita, American Samoa experienced more powerful western influence, leading to a more significant impact on diet and lifestyle shifts and a greater public health impact [8,19]. In a study funded by the National Institutes of Health, American Samoa was found to have the highest rates of diabetes and diabetes-related complications in the world [28]. Overall, 47% of the adult population had diabetes and, alarmingly, the onset of this condition was often during early childhood [28]. Another study reviewing data from 200 countries reported that females in American Samoa had the highest recorded average body mass index (BMI) of 34.8 [29]. Obese or overweight individuals accounted for 93% of the adult population in American Samoa. Resembling diabetes patterns, obesity, and weight issues often began in childhood as well [28]. Overall, 47% of second- and third-grade children were overweight or obese, and this percentage increased to 71.3% of children in the eleventh grade [28]. In Samoa, 85% of the population was obese or overweight [8], and data from 2013 showed that diabetes prevalence was 19.6% in men and 19.5% in women [30].

It is no secret that these chronic diseases are largely preventable, yet the science behind targeting exact lifestyle behaviors, in reference to the physiological origin of chronic diseases, has previously been convoluted and neglected. As understanding of the human body’s reaction to diet, physical activity, and environmental exposure has progressed, the connection has become irrefutable. The consumption of animal products (especially saturated fats in meat and dairy), refined carbohydrates, increased added sugar and salt, and the lack of fiber from decreased fruit and vegetable intake lead to an inflammatory diet that is responsible for creating and accelerating the pathogenesis of chronic diseases, especially diabetes and obesity [31,32].

Nutrition and the pathophysiology of type 2 diabetes and obesity

Insulin and Leptin Insensitivity

The pathogenesis of type 2 diabetes is multifactorial. Increased consumption of processed sugars can lead to insulin resistance and dysfunction. Foods such as white rice, soda, candy, bread, and pastries are quickly digestible and easily accessible forms of carbohydrates, causing sharp spikes in blood glucose which demand insulin [33]. In the traditional Samoan diet, these carbohydrates were locked away with fiber, antioxidants, and phytonutrients within foods such as papaya, bananas, and taro, foods which typically induce satiety, slow digestion, blunt the blood glucose spike, and improve insulin sensitivity and pancreatic function, offering protection against diabetes [19,34-36]. Protein meals can also cause insulin spikes, similar to that of glucose [37,38]. Consumption of animal protein, such as chicken and pork, has increased in Samoa, with pork providing the greatest source of meat calories in Samoa as of 2007 [23]. Additionally, consumption of saturated fats can contribute to this diabetes-inducing diet and peripheral insulin insensitivity, adding to the inflammatory state of the body [33].

Obesity is an inflammatory condition [31]. Just as type 2 diabetes involves desensitization to the hormone insulin, obesity involves desensitization of the body to the hormone leptin (a marker of energy storage). When leptin is released from adipocytes, it binds to the hypothalamus to promote satiety. However, with excess adipose tissue, there are increased leptin levels, which lead to the hypothalamic insensitivity that is characteristic of obesity [39]. Additionally, when saturated animal fat is consumed, it crosses the blood-brain barrier and causes direct damage to the hypothalamus, leading to dysfunction of the appetite-regulating center [40,41]. This indicates that diets high in saturated animal fats have obesogenic effects.

Obesogens

Another culprit for the rise of obesity rates in these islands may be the increased exposure to obesogenic pollutants from canning and packaging processes, antifouling paint, and cooking meats. Globalization has fostered increased import of, and contact with, obesogens in the islands [42]. Bisphenol A, found in canned and processed foods, can promote adipogenesis and is associated with an increased risk of obesity [43]. Phthalates are esters found in meat due to the factory farming and packaging processes that are associated with weight gain [42,44-47]. Tributyltin is a type of organometallic compound that has been used in various industrial marine applications [42,48]. Exposure to this compound through consumption of fish and seafood, together with other “organotins,” activates peroxisome proliferator-activated receptor gamma and results in increased production and size of adipocytes, even in fetal life [49,50]. Due to their negative biological impact, the maritime industry banned the use of organotins in 2008, but recent studies have shown persistent levels in fish samples [51,52]. In 2016, some of these samples “exceeded the good environmental status boundary for tributyltin in seafood” [52]. The highest levels of organotins have been found in halibut, swordfish, and canned tuna (which may have profound implications in American Samoa where many individuals are employed by the Starkist Tuna cannery and where canned tuna is a dietary staple) [53]. Polycyclic aromatic hydrocarbons (PAHs) that can be produced by grilling meat are associated with increased body fat and an increased risk of childhood obesity [54]. Pork has the highest amounts of PAHs compared to other meats and was proportionally the largest source of calories from meat in Samoa [22,55]. Inevitably, global modernization of the food supply involves the increased use of chemical compounds that are both obesogenic and carcinogenic [56]. As the levels of these pollutants are concentrated higher up the food chain, consumption of animal products increases exposure [57-59].

Influence of the Microbiome

The microbiome of individuals has also been found to contribute to the development of obesity. The trillions of bacteria that make up the gut microbiome are largely influenced by diet and play an active role in digestion and metabolism [60]. When diet shifts occur, such as to fewer fruits and vegetables and more meat as in Samoa and American Samoa [9,61], the organisms that make up the microbiome stop helping and start hurting metabolic function [62-64]. Fiber-feeding organisms, when supplied with fiber-rich foods such as taro, breadfruit, and papaya, produce short-chain fatty acids, which are absorbed through the gut wall and help to decrease inflammation, improve metabolism, regulate body fat, and stimulate the production of the appetite-suppressing hormones leptin, peptide YY, and glucagon-like peptide-1 [28,65-68]. Conversely, with increased meat consumption and exposure to carnitine and choline, the bacteria that are fostered in the microbiome can create trimethylamine oxide, which is a marker for increased risk of cardiovascular events and failure to lose weight [69-71]. With continued exposure to diets like these, the microbiomes of individuals change entirely in composition, shifting from predominantly *Prevotella *species (fiber-feeders) to *Bacteroides *species (associated with consumption of animal products) [72,73]. When this shift occurs, there is a loss of the protective effects against weight gain that the plant-predominant diet had fostered in the microbiome [74].

The rise in obesity and diabetes has had a far-reaching impact on the development of nearly all leading causes of chronic diseases and mortality in Samoa and American Samoa, including cancer and cardiovascular diseases [31]. To improve mortality and morbidity and the insurmountable healthcare cost that burdens the islands, immediate action is needed from healthcare professionals to educate themselves and the public on the impact of their diet decisions and the accessible solution of a whole-food, plant-based diet to prevent and reverse chronic diseases. The public health initiatives currently in action in these islands and the suggestions that follow emphasize the health and vitality that is available within Samoa’s rich agriculture and the traditional Samoan diet.

Recommendations: Lifestyle medicine

Lifestyle diseases are difficult to address because of the subtle normalization of toxic lifestyle behaviors over time. These behaviors become so deeply rooted in a society, that in the attempt at prescribing lifestyle modifications as a treatment for the disease, health professionals may be asking individuals to address their culture, customs, and habits [74,75]. However, in both American Samoa and Samoa, the onset of poor food and lifestyle choices has been more recent and rapid than in developed countries such as the United States. At the core of Samoan culture are foods, agriculture, daily activities, and customs that foster vibrant nutrition and health [76]. Therefore, prescribing lifestyle modifications to address the health emergency in these islands involves implementing culturally familiar behaviors. Lifestyle medicine and its powerful effects as a primary treatment approach to the management of chronic disease could relieve substantial death and disability [31]. The medical discipline of lifestyle medicine, ushered in by the American College of Lifestyle Medicine (ACLM), centers on implementing “evidence-based therapeutic approaches, such as eating a predominantly whole food, plant-based diet, getting regular physical activity, adequate sleep, managing stress, avoiding the use of risky substances and pursuing other non-drug modalities, to treat, reverse, and prevent chronic disease” [77]. Although there are several similarities to other conventional medicine, such as preventative, functional, and integrative medicine, lifestyle medicine shifts these evidence-based practices from being adjunct care to being the primary approach to treating the underlying causes of the disease, and medications are used as adjunct care. The lifestyle medicine approach is one in which the physician or provider serves as a guide for the patient to take an active role in their care. Lifestyle medicine highlights the use of a shared treatment model with the inclusion of partnered healthcare professionals to provide much of the direct motivational counseling [78,79]. For Samoa, applying the principles of lifestyle medicine means reinvigorating healthful agricultural practices, cuisine, and physical activity, and bringing new knowledge and awareness to the population.

Plans put in motion by the government, private institutions, and global organizations, including the Samoa Ministry of Health Sector Plan from 2008-2018, the WHO’s Package of Essential NCD interventions adapted to Samoa (PEN Fa’a Samoa), and the Samoa-WHO Country Cooperation Strategy 2018-2022, have unified goals in reducing NCD burden, increasing accessible education on chronic diseases, and encouraging sustainable community accountability for health and well-being [80-82]. With the disproportionate burden that diabetes and obesity have on these polities, targeted research and programs have focused on incorporating culturally appropriate solutions. Programs in Samoa could be improved upon by utilizing the power of lifestyle medicine, most predominantly by a whole-food, plant-based diet. As an example of its potential efficacy, one study showed that switching only 5% of protein from animal to plant sources reduced diabetes risk by 23% [31]. ACLM provides a knowledge base through a curriculum that offers continuing medical education credits and certifications for health professionals, and simple handouts for patients with the up-to-date scientific evidence of the power of a plant-based diet on approaching the leading causes of mortality [83-85]. These resources can be adapted to mirror local agriculture and Samoan plant-based food preparations and can be distributed through a community-based approach to village members. Initiatives such as the Samoa Ministry of Health’s “Grow and Eat Dark Green Leafy Vegetables” [86] embody the goal of incorporating lifestyle medicine into a culturally relevant context. Culturally, in Samoa, family members have a central role in caring for the individual. As these modern diseases have increased in prevalence in these islands, increased dependence on modern medicine has disrupted their roles as caregivers, leading to cultural stress [26]. A reinvigoration of many traditional practices can help resolve social, familial, and cultural stressors that exist due to swift modernization and are associated with changes in immune function and blood pressure, which play into the cyclical aggravation of chronic disease [26,87,88].

## Conclusions

Samoa and American Samoa suffer from profound obesity and chronic NCDs. With morbidity and mortality from chronic diseases on the rise, it is critical that lifestyle factors be addressed. The roles of insulin and leptin insensitivity, obesogens, and the microbiome analyzed in this review are only a few of the many physiological connections between lifestyle decisions and the state of public health. From this analysis, it is suggested that prescribing lifestyle modifications to address the health emergency in these islands is the optimal treatment approach. Individual action by physicians and collective efforts by public health officials is necessary to educate themselves and the public on the impact of their diet decisions and the accessible solution of a whole-food, plant-based diet to prevent and reverse chronic diseases. These lifestyle modifications encourage wellness and foster a return to the vibrant and health-promoting roots of Samoan society.
